# Patterns of sexual behaviour associated with repeated chlamydia testing and infection in men and women: a latent class analysis

**DOI:** 10.1186/s12889-021-12394-0

**Published:** 2022-04-05

**Authors:** Inga Veličko, Alexander Ploner, Lena Marions, Pär Sparén, Björn Herrmann, Sharon Kühlmann-Berenzon

**Affiliations:** 1 Department of Medical Epidemiology and Biostatistics, Karolinska Institutet, P.O. Box 281, Nobels väg 12A, 171 65 Solna, Sweden; 2 Department of Public Health Analysis and Data Management, Public Health Agency of Sweden, Nobels väg 18, 171 82 Solna, Sweden; 3 Department of Clinical Science and Education, Karolinska Institute, Stockholm, Sweden; 4 Section of Obstetrics and Gynaecology, Stockholm South General Hospital, Stockholm, Sweden; 5 Section of Clinical Bacteriology, Department of Medical Sciences, Uppsala University, Uppsala, Sweden; 6 Department of Clinical Microbiology, Uppsala University Hospital, Uppsala, Sweden

**Keywords:** Latent class analysis, Sexual behaviour patterns, Testing for *Chlamydia trachomatis*, Ordered logistic regression, Stratified analysis by sex, Sweden

## Abstract

**Background:**

Adolescents and young adults are at higher risk of acquiring *Chlamydia trachomatis* infection (chlamydia), so testing is promoted in these populations. Studies have shown that re-testing for chlamydia is common amongst them. We investigated how sexual risk behaviour profiles are associated with repeated testing for chlamydia.

**Methods:**

We used baseline data from a cohort of 2814 individuals recruited at an urban STI -clinic. We applied latent class (LC) analysis using 9 manifest variables on sexual behaviour and substance use self-reported by the study participants. We fitted ordered logistic regression to investigate the association of LC membership with the outcomes repeated testing during the past 12 months and lifetime repeated testing for chlamydia. Models were fit separately for men and women.

**Results:**

We identified four LCs for men and three LCs for women with increasing gradient of risky sexual behaviour. The two classes with the highest risk among men were associated with lifetime repeated testing for chlamydia: adjOR = 2.26 (95%CI: 1.50–3.40) and adjOR = 3.03 (95%CI: 1.93–4.74) as compared with the class with lowest risk. In women, the class with the highest risk was associated with increased odds of repeated lifetime testing (adjOR =1.85 (95%CI: 1.24–2.76)) and repeated testing during past 12 months (adjOR = 1.72 (95%CI: 1.16–2.54)). An association with chlamydia positive test at the time of the study and during the participant’s lifetime was only found in the male highest risk classes.

**Conclusion:**

Prevention messages with regard to testing for chlamydia after unprotected sexual contact with new/casual partners seem to reach individuals in highest risk behaviour classes who are more likely to test repeatedly. Further prevention efforts should involve potentially more tailored sex-specific interventions taking into consideration risk behaviour patterns.

**Supplementary Information:**

The online version contains supplementary material available at 10.1186/s12889-021-12394-0.

## Background

Among bacterial sexually transmitted infections (STIs), *Chlamydia trachomatis* infection (chlamydia) has the highest burden globally [1], with the potential to cause serious reproductive health sequalae, such as pelvic inflammatory disease, ectopic pregnancy, and tubal infertility [2–6]. As chlamydia infection is often asymptomatic [7, 8], control measures are aimed at reducing chlamydia incidence and prevalence, as well as potential complications, through screening (testing), treatment and partner notification [9]. Recommendations for annual chlamydia screening in Europe target sexually active individuals under 25 years of age, and those who have had a new sexual partner or more than one partner in the previous year [10]. In the USA, similar recommendations target women, and are extended to young males with high chlamydia prevalence [11]. Repeat testing after initial infection has been found to be beneficial, since repeated chlamydia infections are common [12–14], with recommendations for re-testing of chlamydia positive individuals varying between 3 and 12 months in different countries [10, 11].

Sweden has no restrictions on chlamydia testing; anyone who wishes to be tested has the opportunity to do so. The official recommendation is aimed at persons with a recent new partner or who have had unprotected sexual contact [15]. Testing is based on opportunistic screening (testing) of adolescents and young adults aged 15–29 years, with the intention of increasing testing coverage as part of the National Action Plan for Chlamydia Prevention [16]. The number of reported chlamydia tests increased consistently between 2009 (496522) and 2018 (591460), with chlamydia positivity dropping from 7.6 to 5.4% during the period [17]. Interned-based testing likely contributed to this, accounting for over 20% of all chlamydia tests in 2018 [18].

Independent factors associated with repeated testing were reported elsewhere, that is, younger than 25 years, female sex, co-infection with HIV or gonorrhoea, and increased number of sexual partners during the previous 6 months [19–21]. However, it is reported that risk factors for adverse health conditions co-occur [22]. Similarly, according to the syndemic theory, single sexual behaviours could synergistically interact with other behaviours, such as alcohol and drug use [23–26]. Therefore, classical regression analysis (i.e., variable-oriented), which looks at the association between independent variable and outcome variable while holding other variables constant is not capturing full picture. In contrast, a person-oriented analysis approach, such as latent class analysis (LCA), captures how multiple variables co-occur and interact with each other [27, 28]. This approach allows a multidimensional perspective, where sexual behaviour, substance use, and demographic variables interconnect. It can unmask subgroups (classes) of individuals within the population of interest.

We initiated the present study to gain a better knowledge about population subgroups tested repeatedly for chlamydia to contribute to the improvement of chlamydia prevention. We had two objectives: 1) to identify subgroups (latent classes) based on sexual behaviour and substance use patterns; 2) to study how membership of different latent classes is associated with repeated chlamydia testing and repeated chlamydia infection. Our hypothesis was that members of high-risk behaviour latent classes (LCs) are more likely to test repeatedly and acquire chlamydia repeatedly compared with low-risk behaviour classes.

## Methods

### Study participants

We used data from a published cohort study at an STI-clinic in Stockholm [29]. Visitors aged 20–39 years presenting for chlamydia testing at the clinic between December 2007 and June 2008 were invited to take part in the study. Participants signed a written consent to link their answers in a questionnaire with the result of their test for chlamydia. The questionnaire included topics on sexual behaviour, testing behaviour and experience of substance use (see Table 1S in Online supplement) prior to providing a sample for chlamydia testing [30]. In total 2814 individuals met inclusion criteria and were included in the parent and current study.

### Measures

#### Manifest variables of sexual behaviour and substance use of latent class membership

To identify LCs, we initially selected 12 out of 26 variables related to sexual behaviour and substance use common to men and women. Table 1S in the Online Supplement shows the original manifest variables and our reasoning for the selection for LCA based on the published literature and our expert judgement.

Variables were taken directly from the original questionnaire [30], however, we combined two variables to construct a new variable “Current steady relationship and concurrent sexual contacts during past 12 months” (Table 2S in Online Supplement). Another two variables, originally selected for LCA, were omitted from the final model due to collinearity or low response rate (Table 2S in Online Supplement). Furthermore, we collapsed response categories of some variables included in the LCA, since latent class models were not feasible owing to small counts in some of the initial response categories of the variables (see details in Table 2S with new categories). As a result, nine variables were included in the LCA (Table 1).

**Table 1:** Manifest variables (*n* = 9) for the latent class analysis characterized by sex. The highest risk category item for each variable is highlighted in bold

Manifest variables	Women (***n*** = 1378) (% of column)	Men (***n*** = 1436) (% of column)	Total study population (***N*** = 2814) N (% of column)
**Main reason for current chlamydia testing**			
- Safety and new partner requested and Other	628 (45.6)	643 (44.8)	1271 (45.2)
- **Sex with casual partner**	324 (23.5)	261 (18.2)	585 (20.8)
- Contact with chlamydia case	183 (13.3)	278 (19.4)	461 (16.4)
- Symptoms	237 (17.2)	249 (17.3)	486 (17.3)
- Missing information	6 (0.4)	5 (0.4)	11 (0.4)
**Current steady relationship and concurrent sexual contacts during past 12 months**			
- **No steady partner and no or missing concurrent partners**	817 (59.3)	785 (54.7)	1602 (57.0)
- Yes steady partner and no concurrent partners	205 (14.9)	235 (16.4)	440 (15.6)
- Yes steady and yes concurrent partners	259 (18.8)	272 (18.9)	531 (18.9)
- Yes steady and missing concurrent partners	22 (1.6)	19 (1.3)	41 (1.5)
- Missing information on steady partnership	75 (5.4)	125 (8.7)	200 (7.11)
**Number of sexual partners during the past 12 months**			
- 0-2 partners	332 (24.1)	290 (20.2)	622 (22.1)
- 3-5 partners	613 (44.5)	528 (36.8)	1141 (40.6)
- ≥**6 partners**	303 (22.0)	476 (33.2)	779 (27.7)
- Missing information	130 (9.4)	142 (9.9)	272 (9.7)
**Type of the last sexual partner**			
- Steady partner	428 (31.1)	396 (27.6)	824 (29.3)
- Recurrent partner	411 (29.8)	341 (23.8)	752 (26.7)
**- Casual unknown partner**	111 (8.1)	174 (12.1)	285 (10.1)
**- Casual known partner**	138 (10.0)	141 (9.8)	279 (9.9)
- Other type	116 (8.4)	111 (7.7)	227 (8.1)
- Missing information	174 (12.6)	273 (19.0)	447 (15.9)
**Condom use with new/ casual partners**			
- **Never and seldom**	383 (27.8)	460 (32.0)	843 (30.0)
- Often and always	987 (71.6)	969 (67.5)	1956 (69.5)
- Missing information	8 (0.6)	7 (0.5)	15 (0.5)
**Taking responsibility for obtaining condom**			
- **Never and often not**	341 (24.8)	221 (15.4)	562 (20.0)
- Sometimes	668 (48.5)	620 (43.2)	1288 (45.8)
- Always	362 (26.3)	589 (41.0)	951 (33.8)
- Missing information	7 (0.5)	6 (0.4)	13 (0.5)
**Alcohol use before having sex (past 6 months)**			
- No	133 (9.7)	110 (7.7)	243 (8.6)
- Sometimes	457 (33.2)	388 (27.0)	845 (30.0)
- **Several time and don’t remember/don’t know**	783 (56.8)	930 (64.8)	1713 (61.0)
- Missing information	5 (0.4)	8 (0.6)	13 (0.4)
**Alcohol impact on taking higher sexual risks than expected by respondent**			
- No and little impact	538 (39.0	520 (36.2	1058 (37.6)
- Some impact	482 (35.0)	531 (37.0)	1013 (36.0)
- **Big impact and don’t remember/don’t know**	204 (14.8)	249 (17.3)	453 (16.1)
- Not applicable, did not drink	133 (9.7)	110 (7.7)	243 (8.6)
- Missing information	21 (1.5)	26 (1.8)	47 (1.6)
**Drug use before having sex (past 6 months)**			
- No	1246 (90.4)	1264 (88.0)	2510 (89.2)
- **Any use and don’t remember/don’t know**	116 (8.4)	161 (11.2)	277 (9.8)
- Missing information	16 (1.2)	11 (0.8)	27 (1.0)

#### Demographic and sex-specific variables across latent classes

We described the probabilities resulting from the LCA for covariates common to men and women and for covariates specific to each sex. Common covariates were age group and marital status, while sex-specific covariates were “Got woman unintentionally pregnant” for men; and for women, “Use of contraception method”, “Use of emergency contraceptive pills”, and “History of induced abortion”.

#### Distal outcomes

We investigated the association between LCs and two outcomes: testing and being infected with chlamydia. For each outcome, we looked at short-term and long-term measures (Table 2). For short-term testing, we looked at repeated testing for chlamydia during the past 12 months (no/yes). For long-term testing, we analysed repeated lifetime testing for chlamydia (no; 1–3 times; four or more times). Correspondingly, for chlamydia infection short-term, we looked at current chlamydia test results at the time of recruitment (negative/positive), and for long-term outcomes, repeated lifetime chlamydia infection (never; once; twice or more times). No and never were considered as reference levels in all outcome analyses.

**Table 2:** Distal outcome variables

Outcome variables	Women (n = 1378) (% of column)	Men (n = 1436) (% of column)	Total study population (N = 2814) N (% of column)
**Short-term testing outcome: Chlamydia testing during the past 12 months**			
- Yes	715 (51.9)	494 (34.4)	1209 (42.9)
- No	534 (38.8)	642 (44.7)	1176 (41.8)
- Don’t remember and missing information^a^	129 (9.4)	300 (20.9)	429 (15.2)
**Long-term testing outcome: Lifetime testing for chlamydia**			
- Never	103 (7.5)	350 (24.4)	453 (16.1)
- 1-3 times	875 (63.5)	862 (60.0)	1737 (61.7)
- ≥4 times	375 (27.2)	198 (13.8)	573 (20.4)
- Don’t remember and missing information^a^	25 (1.8)	26 (1.8)	51 (1.8)
**Short-term infection outcome: Present laboratory verified chlamydia infection**			
- Yes	122 (8.8)	181 (12.6)	303 (10.8)
- No	1256 (91.2)	1255 (87.4)	2511 (89.2)
**Long-term infection outcome: Self-reported lifetime chlamydia infection**			
- Never	805 (58.4)	712 (49.6)	1517 (53.9)
- Once	370 (26.9)	317 (22.1)	687 (24.4)
- ≥2 times	110 (8.0)	92 (6.4)	202 (7.2)
- Missing information and don’t remember^a^	93 (6.8)	315 (21.9)	408 (14.5)
**Age group (considered as confounder)**			
- 20–24	465 (33.7)	367 (25.6)	832 (29.6)
- 25-29	583 (42.3)	626 (43.6)	1209 (42.9)
- 30-34	219 (15.9)	304 (21.2)	523 (18.6)
- 35-40	111 (8.1)	139 (9.7)	250 (8.9)

^a^ Categories “Missing information and don’t remember” were collapsed and excluded from the latent class analysis

Due to differences in sexual behaviour, we carried out the analyses for each sex independently, and we adjusted regression models for age group (20–24, 25–29, 30–34 and 35–40 years; with the latter as a reference level).

#### Statistical analyses

Latent class models with varying numbers of LCs (2–6) were fitted, based on the observed 9 manifest variables (Table 1). We selected the number of LCs based on the minimal or close to minimal Akaike Information Criterion (AIC) and Bayesian Information Criterion (BIC), numerical convergence and stability of the model fit, as well as on differential interpretation of competing models. We also calculated the entropy for each LC model, where values approaching one indicate clearer separation between latent classes [31]. The conditional response probabilities and LC prevalence were estimated using the maximum likelihood criterion. Each respondent was assigned to the LC with estimated highest latent class probability.

For interpretation and labelling LCs, we first identified for each manifest variable the response category carrying the highest risk for sexually transmitted infections (STIs). For example, for the manifest variable “Steady and Concurrent relationship” we chose the category “No steady partner and no/missing concurrent” as our highest risk category. Based on the estimated probabilities for each identified category of the variable we chose labels for the LCs (see details in Table 3S–4S in Online Supplement).

**Fig. 1:**
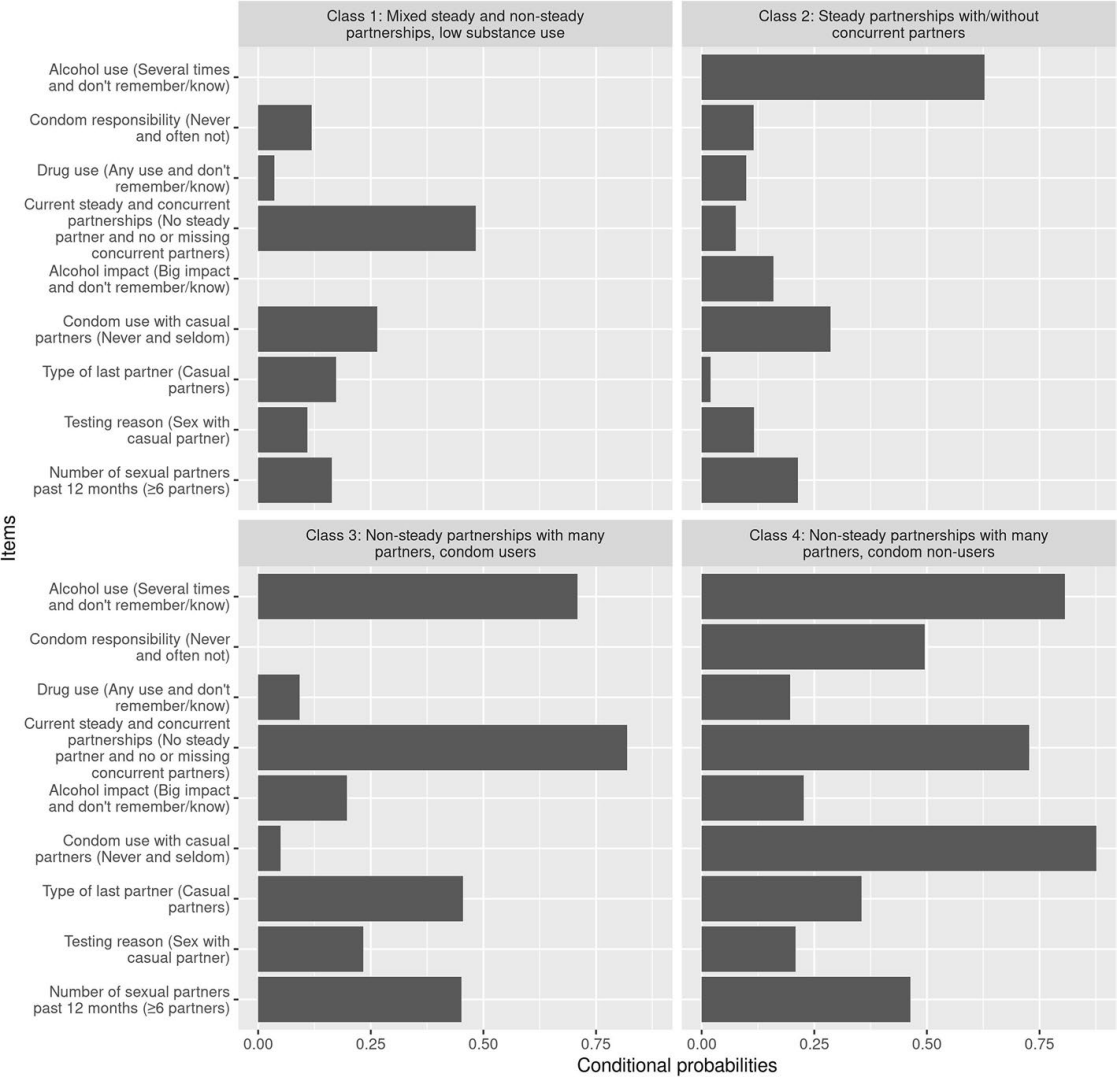
Latent class conditional probabilities for men (N = 1436), presented as probabilities of the highest risk category item for each variable. The most discriminatory items are at the top of the panel and sorted by entropy

**Fig. 2:**
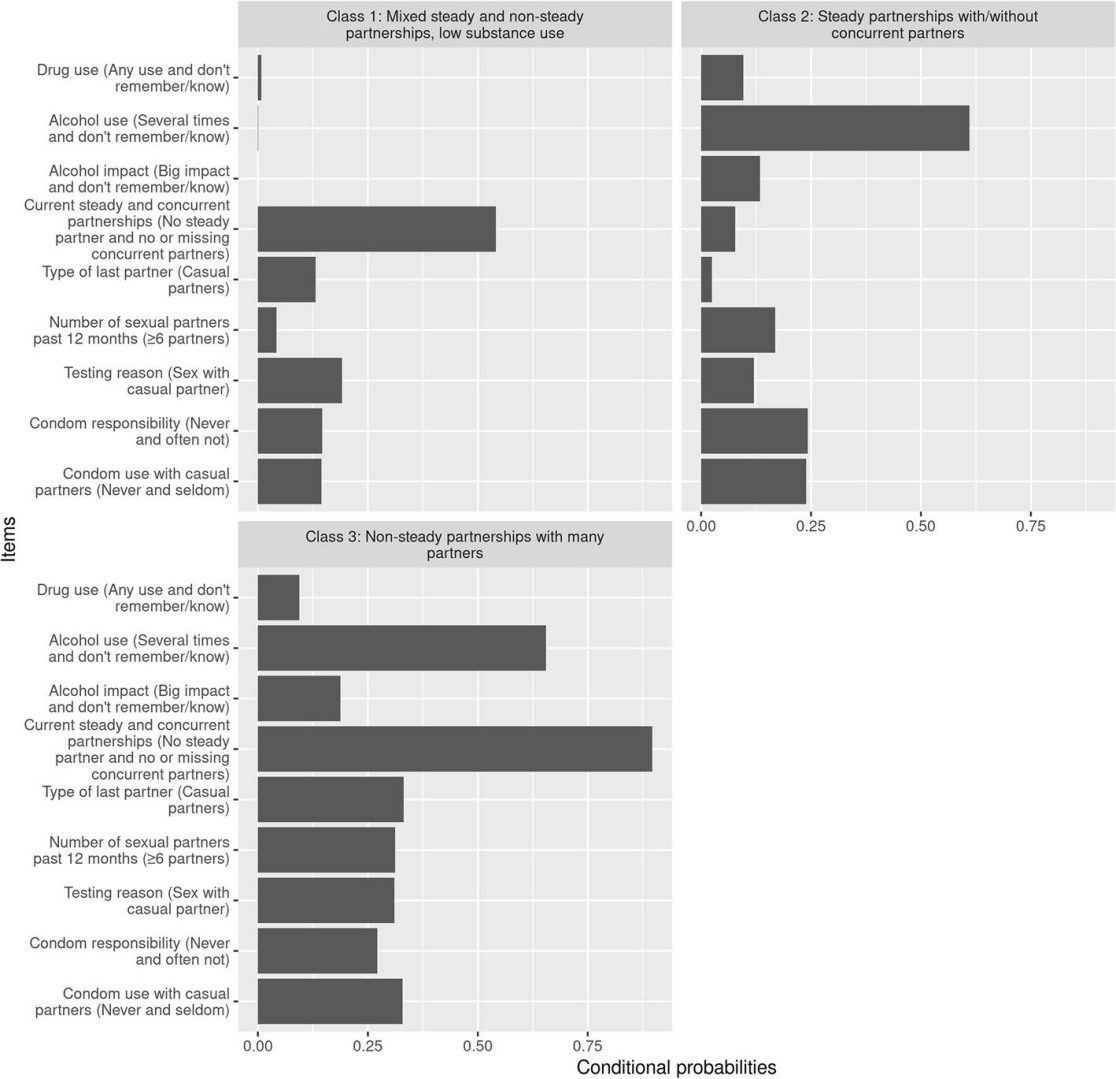
Latent class conditional probabilities for women (N = 1378), presented as probabilities of the highest risk category of each variable. The most discriminatory items are at the top of the panel and sorted by entropy

**Fig. 3:**
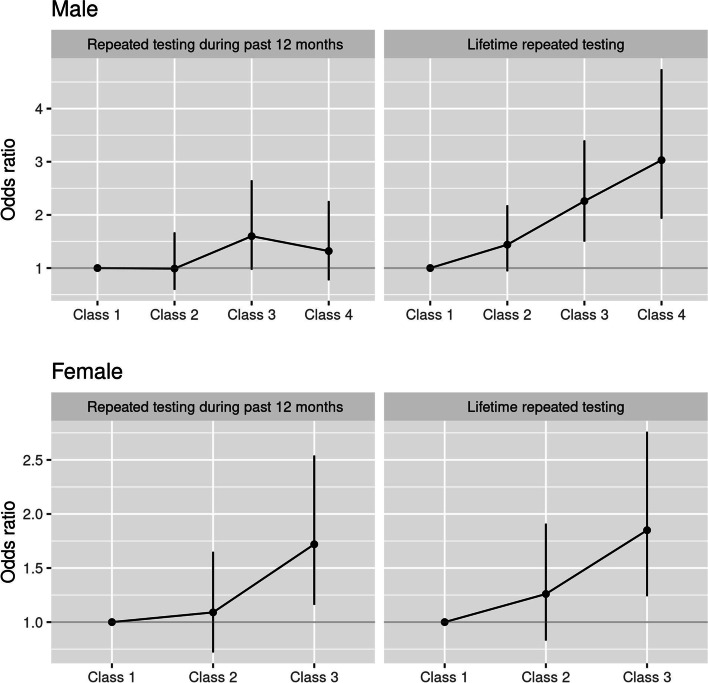
Association between latent class membership and repeated testing by sex, adjusted for age groups. All results from proportional odds logistic regression models

**Fig. 4:**
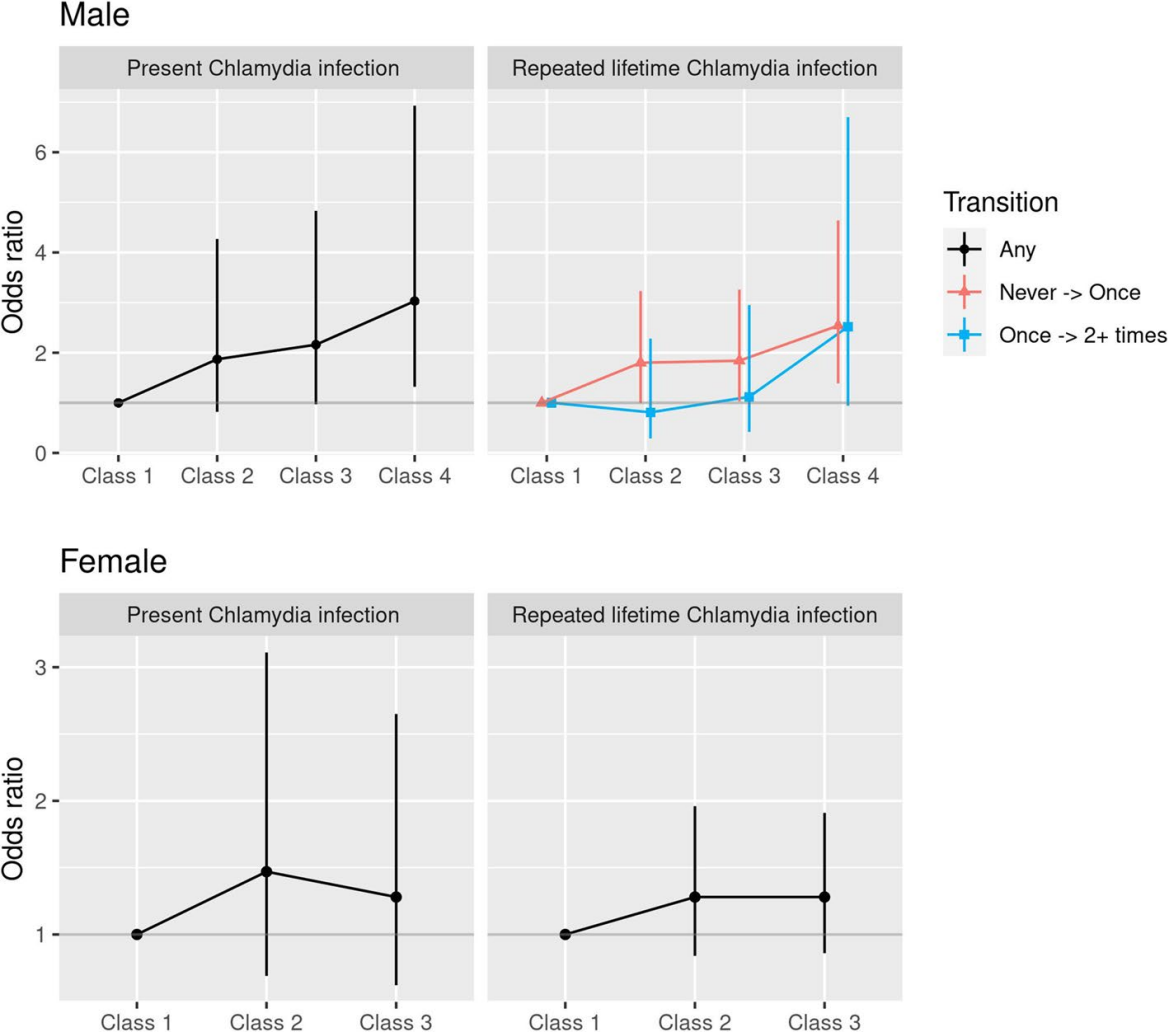
Association between latent class membership and lifetime chlamydia infection by sex, adjusted for age groups. All results are from proportional odds logistic regression models (same relationship between latent classes and categories of the outcome). The only exception is the relationship between latent classes and Repeated lifetime Chlamydia infection in men, where the results from the multinomial logistic regression model are presented with varying relationships between LCs and categories of the outcome (Never-- > Once, Once-- > Twice or more times)

We ordered LCs according to sexual risk-behaviour for general STIs (see Table 1S for references) by considering only the same highest-risk category of each manifest variable, as used for the labelling of the LCs (see above). Thus, Class 1 comprised individuals with the lowest probabilities of highest-risk sexual behaviour and substance use (e.g., number of sexual partners 6 or more during past 12 months, alcohol use several times), which we considered as class of “lowest-risk behaviour”, and used as a reference level in all analyses. The LCs with highest probabilities of high-risk sexual behaviour and substance use were considered as “highest-risk behaviour” classes (Class 3 and 4). We assigned the remaining LC (Class 2) to the “moderate-risk behaviour” LC, since probabilities of highest-risk sexual behaviour and substance use were in between “lowest-risk behaviour” and “highest-risk behaviour” classes; see Figs. 1, 2, 3 and 4 where we present LCs in the ascending order of risk behaviour as we defined above.

We assessed the association between LC membership and distal outcomes via regression models. For the dichotomous short-term outcomes (repeated testing during 12 months, chlamydia infection at current test occasion), we fitted ordinary logistic regression models with the LCs as predictor variable, adjusted for age group. For the three-level long-term outcomes (lifetime testing for chlamydia, lifetime chlamydia infections), we first fitted proportional odds ordinal logistic regression models, again with the identified LCs as independent predictor variable and adjusted for age group [32]. The main assumption in this model was that the relationship between all categories of the outcome is the same, i.e. proportional. This model produces one set of adjusted odds ratios that describe the relative odds of both the intermediate outcome level vs the lowest outcome level, and the highest outcome level vs the intermediate outcome level. We then tested the assumption of proportional odds via a Brant test [33]. For the outcome “Lifetime chlamydia infection” in men, we found significant evidence that the assumption was violated, and we consequently re-fit this as a multinomial (polytomous) logistic regression model instead; this model generates two sets of odds ratios, one for the intermediate vs lowest outcome level comparison, and one for the highest vs intermediate outcome level comparison [34]. For the long-term distal outcomes, we also performed a linear trend test (Wald test). We reported adjusted odds ratios (adjORs) with 95% confidence intervals (CIs).

We used Stata v. 15 for all analyses [35] and used R statistical software to produce figures [36].

## Results

### Study participants

We recruited 2814 individuals, of whom 1436 (51%) were men [29]. The age of the respondents was 20 to 40 years, with a mean age for women of 27.0 (± 4.3) years and a mean age for men of 27.8 (± 4.4) years. Two thirds of men and women were single [29].

### Latent classes by sex

Based on the nine selected manifest variables, we fitted models with two to five LCs for men and up to three LCs for women (models with more classes did not converge). Model AIC and BIC values strongly supported three classes for women, and provided strong evidence for either four or five classes for men (Online supplement Table 5S). Closer comparison of the two candidate models for men revealed some numerical instability, a less interpretable solution (not shown) and a lower entropy for the five-class model, which led us to adopt the four-class model for men (Fig. 1, Online supplement Table 5S).

We interpreted, labelled, and ordered the LCs based on the item-response probabilities (Online supplement Table 3S–4S), with Class 1 representing the least risky behaviour, and Class 4 for men and Class 3 for women the riskiest behaviour. We present the probabilities of the highest risk category of each manifest variable in Figs. 1 and 2 as support for this characterization, For men, 8% (*n* = 110) fell into Class 1 (lowest-risk behaviour class), labelled “Mixed steady and non-steady partnerships, low substance use”, characterized by highest probability of reporting steady partnerships, with higher probability of reporting 0–2 sexual partners, no alcohol use and very low probability of drug use (Table 3S, Fig. 1). Thirty percent (*n* = 441) of men fell into Class 2 (moderate-risk behaviour), labelled “Steady partnership with/without concurrent partners” which was characterized by the highest probability of reporting steady partnerships, alongside with equal probability of having/not having concurrent relationships, lower probability of reporting ≥6 sexual partners during the past 12 months, with high probability using condoms “often and always” with casual partners, with relatively high probability of using alcohol and low use of drugs. For men, we could further separate LCs of highest-risk behaviour: “Non-steady partnerships with many partners, condom users” (Class 3, *n* = 601) and “Non-steady partnerships with many partners, condom non-users” (Class 4, *n* = 284). These LCs contained 42 and 20% of the men, respectively (Table 3S, Fig. 1). These two classes were similar in their probabilities of reporting high probability of not having steady partnerships, higher probability reporting ≥6 sexual partners during past 12 months, high probability of alcohol use. The only distinguishing features were: difference in condom use “never and seldom” with casual partners (low for Class 3 and high for Class 4), which reflected also in the responsibility for condoms, and difference in drug use (low for Class 3 and higher for Class 4) (Table 3S, Fig. 1).

Among women, similar latent risk classes were observed, also in terms of size, as for men. Among women, 10% (*n* = 134) fell into Class 1 (lowest-risk behaviour), labelled “Mixed steady and non-steady partnerships, low substance use” and characterized by almost equal probability of reporting steady and non-steady relationships, lowest probability of reporting ≥6 sexual partners during past 12 months, lowest probability of reporting condom use “never and seldom” with casual partners, lowest probability of alcohol and drug use (Online supplement Table 4S, Fig. 2). Thirty-two percent (*n* = 441) of women fell into Class 2 (moderate-risk behaviour), labelled “Steady partnership with/without concurrent partners” characterized by highest probability of reporting steady partnerships alongside with equal probability of having/not having concurrent relationships, lower probability reporting ≥6 sexual partners during past 12 months, with higher probability using condoms “never and seldom” with casual partners, with relatively high probability of using alcohol and higher use of drugs (Table 4S, Fig. 2). The largest Class 3 (highest-risk behaviour), containing 58% (*n* = 803) of women, was labelled “Non-steady partnerships with many partners”, and was characterised by a high probability of having a non-steady current partner and a higher probability of having 6 or more sexual partners during the previous 12 months compared with the other female LCs. The probability of frequent alcohol use before sex was high among both women and men across all LCs, with the exception of Class 1.

### Demographic and sex-specific variables across latent classes

Class membership was similar amongst the men and women across the age groups and marital status (Online supplement Table 6S – 7S). Notably, the younger (20–29 years of age) men (76%) and women (77%), and single men (88%) and women (96%) were more likely to belong to high-risk classes (Class 4 and 3, respectively). The men in Class 4 were also more likely (40%) to impregnate women unintentionally than men in other LCs. The absolute majority (80–87%) of women used some type of contraception across LCs. However, women in Class 3 were more likely to use the barrier method (35%). There was no major difference in the use of emergency contraceptive pills or a history of induced abortion across LCs.

### Distal outcomes

#### Short-term outcome: repeated testing during past 12 months and current chlamydia infection

For repeated testing for chlamydia during past 12 months, we found significantly higher odds of 1.72 (95%CI: 1.16–2.54) in highest-risk behaviour Class 3 compared with Class 1 (Fig. 3, Online supplement Table 8S) among women. Among men, there was a borderline statistically non-significant association with highest-risk behaviours Class 3, adjOR = 1.60 (95%CI: 0.97–2.65), Fig. 3, Online supplement Table 9S.

Among men, Class 4 had 3.03 (95%CI 1.32–6.93) times higher odds than Class 1 of testing positive for the current chlamydia infection (Fig. 4, Online supplement Table 10S). Class 3 in men had borderline statistically non-significant increased odds as well: adjOR = 2.16 (95%CI: 0.97–4.83). None of the associations were statistically significant for this outcome among women (Fig. 4, Online supplement Table 11S).

#### Long- term outcome: repeated lifetime testing and repeated lifetime chlamydia infection

Both the highest-risk male classes and the highest-risk female class were all significantly associated with at least a two-fold increased odds of repeated lifetime testing (Fig. 3, Online supplement Table 8S–9S). Among men, Class 3 had an adjOR = 2.26 (95%CI: 1.50–3.40), while Class 4 had an even stronger association with adjOR =3.03 (95%CI: 1.93–4.74). Among women, we estimated 1.85 (95%CI: 1.24–2.76) higher odds of repeated lifetime testing in the highest-risk Class 3 compared to Class 1. We found a statistically significant linear trend for this outcome in both men and women, which indicated a dose-response relationship: increasing levels of risk behaviour LCs were associated with increased odds of repeated lifetime testing for chlamydia.

In contrast to the results presented above, we found that for men, the relationship between LCs varied between outcome levels (never, once, twice or more) of repeated lifetime chlamydia infection (Fig. 4, Table 10S in Online Supplement). For a comparison between outcome categories “once” versus “never”, we found an approximately linearly increasing trend across LCs, with Class 3 having 1.84 (95%CI: 1.03–3.26) higher odds than Class 1 of having had one previous chlamydia infection, and Class 4 having 2.54 (95%CI: 1.39–4.64) higher odds compared to Class 1 (red line in the corresponding panel in Fig. 4). In contrast, the odds of having chlamydia twice or more compared with having had it only once was not increased for Classes 2 and 3, and the increased odds for Class 4 were not statistically significant (OR = 2.52, 95%CI: 0.94–6.70) (blue line in the corresponding panel in Fig. 4, Online supplement Table 10S). Among women, none of the associations were statistically significant for lifetime repeated chlamydia infection (Fig. 4, Online supplement Table 11S).

## Discussion

In a large cohort of visitors to the STI-clinic, we identified LCs, which represented a diversity of sexual behaviour, and ranged from lowest- to highest risk sexual behaviour. Our result showed that sexual behaviours and substance use co-occur and are associated with repeated testing for chlamydia during their lifetime for both sexes and with repeated testing during the past 12 months among women. The men in the highest-risk latent classes had a two-fold higher odds of being infected once during their lifetime and a three-fold higher odds of having a current chlamydia infection. No associations between LC membership and chlamydia infection were found amongst the women.

We identified four distinct LCs for the men and three LCs for the women. The majority (60%) of respondents of both sexes fell into highest-risk behaviour LCs, which may have been expected given that the entire cohort was recruited at an STI-clinic, where a higher proportion of individuals with high-risk behaviour are more likely to be presented, as has been noted elsewhere [37–39]. For both sexes we saw similarities in important discriminators of class profiles, such as pre-sex alcohol use and use of other drugs (cannabis the most frequently cited). Pre-sex alcohol use can lead to poor judgement on sexual partner choice (e.g., casual partner), an increased number of sexual partners, condomless sex, and regrets about having had sex as was reported in other studies [40–42]. Additionally, other studies have suggested that people who fail to use condoms after drinking possibly also fail to use them when they abstain from drinking; thus, such behaviour is believed to be more likely related to personality traits [43, 44]. Combined substance use of drugs and alcohol is reported to be clustered together [45, 46] with the purpose to facilitate sexual contact and to enhance the sensations of sexual intercourse has been described previously [40]. The variable Type of current sexual partnership (steady vs casual) was also strong discriminator of the profiles both men and women and is reported elsewhere to vary in condom use [47]. Less successful discriminators in our class profiles were condomless sex with casual partners and number of sexual partners during the previous 12 months. However, several earlier studies have reported that respondents consider it important to use condoms and have the intention to use them, but actual use varies with the type of partner and the form of sexual contact [47–51]. This was reflected amongst the men in our study, where further separation of the high-risk classes was possible: one class was described as condom users (Class 3) and the other non-condom users (Class 4). An increased number of sexual partners is known independent risk factor for chlamydia [29, 52, 53] and was one of the discriminating variables in women. In our LCA, however, we found that this also co-occur with decreased condom use in highest- and moderate-risk behaviour LCs. The moderate-risk sexual behaviour class was also characterised by a high probability of concurrent (casual) partnerships, despite a high proportion of current steady partnerships. These results from our study were consistent with previous LCA studies where these factors were a significant facilitator of STI acquisition [54–57]. These identified similarities and differences in the profiles of men and women in our cohort have implications for the different approach towards these populations, which we also explored further.

We found that individuals of highest-risk classes of both sexes had a higher odds of being tested repeatedly, which supported our hypothesis. Studies have shown consistently that repeated testing may facilitate short-term change in high-risk behaviour if individuals receive positive chlamydia results [58, 59] but not negative results [60], suggesting that testing has unintended consequences [61–63]. Furthermore, a recent study suggested that young adults who engage in unsafe sex possibly have repeated tests for chlamydia as a replacement for condom use [64]. Repeated testing for chlamydia in highest-risk classes in our study suggest that members of these LCs had absorbed Swedish public health messages to test for chlamydia after unprotected sexual contact with a new or casual partner [16]. Recent study in Stockholm County reported (after controlling for social-economic factors and previous positive chlamydia test) that actually 42% of young people had tested repeatedly for chlamydia within a 3-year period [19].

Furthermore, our results also showed that relationship between latent classes and chlamydia infection differed by sex. Men in the highest-risk classes were more likely to test positive for present chlamydia and at least once during their lifetime as well as test repeatedly, which suggests that they did not change their sexual behaviour. Repeated testing after chlamydia infection due to unchanged risky behaviour has been reported elsewhere [19, 65–67]. Notably, another LCA study reported similar findings to ours that casual sex risk-takers (which is a feature of our latent Class 3 and 4) were more likely to contract STIs [23, 68]. Conversely, we found increased odds amongst the women for LCs 2 and 3 but not statistically significant with effect size smaller than for men. Possible reasons for that could be more consistent condom use in women than in men: in our LCA condom use variable was a better separator of LCs among men (especially Class 3 and 4) but less discriminatory in LCs for women (Figs. 1 and 2, where the most discriminatory items are at the top of the panel). Alternatively, difference in positivity by sex could be partially explained by the difference in testing pattern. Women have more encounters with health care (e.g., routine gynaecology visits, family planning counselling etc.) and therefore have better possibilities for screening for chlamydia and other STIs, while men reportedly have poorer test-seeking behaviour [18, 19].

Accessible testing for chlamydia in Sweden is well accepted by the users [64, 69]. However, it has been argued that introducing a screening program for chlamydia in low-risk populations, where many individuals test negative and might therefore change their sexual behaviour in the direction of greater risk, could hamper screening efforts [60]. As a result, a high prevalence of repeated chlamydia infections is maintained amongst men and women [70, 71]. Furthermore, possible scaling down of testing towards only symptomatic was suggested recently [72]. Our results indicated that risky sexual behaviour (e.g., condomless sexual contacts with casual partners, and higher numbers of sexual partners) were still at high levels amongst the men and at moderate levels for women in the highest-risk classes (Class 4 and 3, respectively), suggesting that the response to interventions might be different in each latent class. Thus, continuous condom promotion is needed as condoms are effective in reducing the risk of chlamydia and other STIs [73], and can reduce chlamydia prevalence substantially [74, 75]. Additionally, alcohol use was highly prevalent amongst our study participants, and therefore efforts to increase condom use could be combined with interventions to decrease alcohol use; this might encourage condom-related protective behavioural strategies in individuals [43, 76].

Our study has several strengths. Firstly, to the best of our knowledge, the present study is the first to associate sexual and substance use risk-behaviour LC membership with repeated testing for chlamydia. Additionally, our LCA was reinforced by the large sample size based on the detailed questionnaire data and the distinction it drew between the sexes. Our study has several limitations. One of the major limitations is that the data was collected in 2008 and might not reflect current behaviours or patterns of behaviours in the population of interest. Nevertheless, the subsequent studies over the years in Sweden in similar STI clinic populations [77] and users of internet-based testing [78] reported congruous independent risk behaviours associated with chlamydia infection. In addition, sexual and substance use behaviours neither changed significantly over the time in the general population [79, 80]. However, we should be careful regarding the fact whether latent class patterns nowadays would look similar to our identified LCs even if based on similar risk factors. Thus, the extrapolation of our results on LCs on current populations should be done with assumption that similar LCs are formed among individuals with the same risk factors as in our study. Another limitation is that our analysis relies on an accurate selection of observed variables to identify latent classes. Additionally, recall bias and self-report bias are common in studies based on self-reported data. Another limitation is that our study population was not randomly sampled from the general population; the fact that they were visitors at an STI-clinic suggests selection bias. Furthermore, we used different recall times for exposure (6 months and 12 months) and outcomes (12 months and across lifetime), which may have biased the observed associations. However, a recent LCA study in a similar setting reported that the majority of its population remained in the same LC for up to one year, which was an indication of relatively stable sexual behaviour [37]. Finally, no causal inference can be drawn from the present study because of potential unmeasured confounding and a lack of temporality.

## Conclusions

In conclusion, we supported our hypothesis that LCs of highest-risk sexual behaviour were associated with the repeated lifetime testing for chlamydia (amongst both sexes) and repeated testing during the previous 12 months (amongst the women). This suggested that public health messages regarding STI testing were being acted on. However, borderline association with repeated chlamydia infection in men of highest-risk classes suggests that they are at risk for STIs and future research should focus on effective interventions to reach these population subgroups. This analysis should be repeated on more recent data, which might provide further insight into current risk behaviour patterns and prevention needs. Our results suggest that efforts at prevention of safe sex should be stepped up with potentially more tailored sex-specific interventions and addressing different risk behaviour patterns.

## Supplementary Information

**Additional file 1.** Contains results of latent class identification, probabilities of latent class memberships by covariates and tables with associations between latent class membership and outcomes.

## Data Availability

The data that support the findings of this study are available from Public Health Agency of Sweden but restrictions apply to the availability of these data, which were used under license for the current study, and so are not publicly available. Data are however available from the authors upon reasonable request and with permission of Public Health Agency of Sweden.

## Abbreviations

AIC: Akaike Information Criterion
adjOR: Adjusted Odds Ratio
BIC: Bayesian Information Criterion
CI: Confidence interval
LC: Latent class
LCA: Latent class analysis
STI: Sexually transmitted infection
