# Supplementary material for: Patterns of sexual behaviour associated with repeated chlamydia testing and infection in men and women: a latent class analysis
Source: BMC Public Health. 2022 Apr 5;22:652. doi: 10.1186/s12889-021-12394-0 (PMC8981706; doi:10.1186/s12889-021-12394-0)
Supplement: Supplementary file 1 — Additional file 1. Contains results of latent class identification, probabilities of latent class memberships by covariates and tables with associations between latent class membership and outcomes. [file 12889_2021_12394_MOESM1_ESM.docx]

Online Supplement Material

**Patterns of sexual behaviour associated with repeated chlamydia testing and infection in men and women: a latent class analysis**

Inga Veličko, Alexander Ploner, Lena Marions, Pär Sparén, Björn Herrmann, Sharon Kühlmann-Berenzon

**Table 1S. Original variables* considered in the latent class analysis**

| **Original manifest variable** | **Reasoning for variable selection or rejection for latent class membership (including references)** |
| --- | --- |
| Age | Included as known confounder [1-6] |
| Marital status | Included into characterization of the demographic differences between latent classes [2, 5] |
| Occupation | Not included as not considered sexual risk behaviour variable |
| Having children | Not included as not considered sexual risk behaviour variable |
| Reason for current chlamydia testing | Included as previously reported risk factor [7] |
| Presenting symptoms at clinic visit | Not included |
| HIV testing (lifetime) | Not included, since is correlated to chlamydia testing variables[8] |
| Chlamydia infection during the past 12 months | Not included due to small sample size and missing data |
| Any lifetime STI | Not included due to small sample size |
| Current steady relationship | Included as previously reported risk factors [4] |
| Concurrent sexual contacts (past 12 months) | Included as previously reported risk factors [4] |
| Number of sexual partners during the past 12 months | Included [1, 2, 4, 7, 5] |
| Number of casual sexual partners (past 12 months) | Not included, since correlated with “Number of sexual partners during the past 12 months” |
| Time since the last sexual contact | Not included, does not belong to long-term sexual behaviour. We wanted to capture long-term behaviour in relation to testing. |
| Type of the last sexual partner | Included [4] |
| Type of the last sexual contact | Initially included [8], but omitted later due to collinearity problems in the model. Additionally, majority of the cohort reported having had vaginal sex. |
| Condom use with new/ casual partners | Included [1, 9, 5, 6] |
| Taking responsibility for obtaining condom | Included as a proxy of risk-taking behaviour |
| Alcohol use before having sex (past 6 months) | Included as a proxy of risk-taking behaviour [5] |
| Alcohol impact on taking higher sexual risks than expected by respondent (n=2 534)** | Included as a proxy of risk-taking behaviour |
| Drug use before having sex (past 6 months) | Included as a proxy of risk-taking behaviour [5] |
| Drug impact on taking higher sexual risks than expected by respondent (n=268)** | Included as a marker of risk-taking behaviour, but omitted later due to low response rate and high correlation with variable “Drug use”. |
| For men only (n=1436):  Have ever made woman unintentionally pregnant | Included into characterization of the demographic differences between latent classes |
| For women only (n=1378):  Current contraception method | Included into characterization of the demographic differences between latent classes |
| Use of emergency contraceptive pills (lifetime) | Included into characterization of the demographic differences between latent classes (lack of contraception use incl., condom non-use). |
| Did induced abortion (lifetime) | Included into characterization of the demographic differences between latent classes as proxy of risk behaviour (lack of contraception use incl., condom non-use). |

***-** Original questionnaire was published elsewhere [10].

** - Follow-up question if answered previous question.

**Table 2S. Variables (with categories) included in latent class analysis and characterization of latent classes**

| **Original manifest variable and its categories** | **Variable and its categories included in LCA** | **Comments** |
| --- | --- | --- |
| **Age** | **Age group** |  |
| - Age calculated from year of birth | - 20-24 years old |  |
|  | - 25-29 years old |  |
|  | - 30-34 years old |  |
|  | - 35-40 years old |  |
| **Marital status** | **Marital status** |  |
| - Married | - Married or cohabitation |  |
| - Cohabitation |  |  |
| - Living apart | - Living apart |  |
| - Single | - Single |  |
| - Other | - Other |  |
| **Reason for current chlamydia testing** | **Reason for current chlamydia testing** |  |
| - Casual sex/ check-up | - Safety and new partner requested and Other |  |
| - Other reason |  |  |
| - Casual sex / check-up | **-** Sex with casual partner |  |
| - Contact with chlamydia case | **-** Partner has CT |  |
| - Symptoms | - Symptoms |  |
| **Current steady relationship** | **Current steady relationship and concurrent sexual contacts during past 12 months**  - No steady partner and no or missing concurrent partners  - Yes steady partner and no concurrent partners  - Yes steady and yes concurrent partners  - Yes steady and missing concurrent partners  - Missing steady | Variable “Current steady relationship and concurrent sexual contacts during past 12 months” was created from two separate variables “Current steady relationship” and “Concurrent sexual contacts (past 12 months)”. The latter question was only applicable and relevant for those who answered “Yes” on the former question. Therefore, in the new collapsed variable, we separated those who did and did not have a current steady partner; those who did not have a current steady partner could not have concurrent partnership by design. Those who had reported steady partner could answer on the following question on concurrent partnership: yes, no and did not answer (missing information) that formed three new categories for those who had a steady partnership. The last category “Missing steady” included those who did not report a current steady partner (missing info), or reported no current steady and yes/no for concurrent partnership, which is not relevant since we do not have information on the steady partner. |
| - Yes |  |  |
| - No |  |  |
| **Concurrent sexual contacts (past 12 months)** |  |  |
| - Yes |  |  |
| - No |  |  |
| **Number of sexual partners during the past 12 months**  - reported number by respondent | **Number of sexual partners during the past 12 months**  - 0-2 partners  - 3-5 partners  - ≥6 partners | 2 persons reported “0 partners” and we kept them in the analysis. |
| **Type of the last sexual partner** | **Type of the last sexual partner** | The definitions of the partner type were provided in the original questionnaire [10]. |
| -Steady partner | -Steady partner |  |
| - Recurrent partner | - Recurrent partner |  |
| - Casual unknown partner | - Casual unknown partner |  |
| - Casual known partner | - Casual known partner |  |
| - Several partners simultaneously | - Other type |  |
| - Other type |  |  |
| **Condom use with new/ casual partners** | **Condom use with new/ casual partners** |  |
| - Never | - Never and seldom |  |
| - Seldom |  |  |
| - Often | - Often and always |  |
| - Always |  |  |
| **Taking responsibility for obtaining condom** | **Taking responsibility for obtaining condom** |  |
| - Never | - Never and often not |  |
| - Often not |  |  |
| - Sometimes | - Sometimes |  |
| - Always | - Always |  |
| **Alcohol use before having sex (past 6 months)** | **Alcohol use before having sex (past 6 months)** | We collapsed category “Don´t remember/don´t know” with “Several times” as we reasoned that the respondent, who did not remember or did not know whether they used alcohol most likely used it. We had 24 persons in the category “Don´t remember/Don´t know “. |
| - No | - No |  |
| - Sometimes | - Sometimes |  |
| - Several times | - Several time and don´t remember/don´t know |  |
| - Don´t remember/Don´t know |  |  |
| **Alcohol impact on taking higher sexual risks than expected by respondent (n=2 534)*** | **Alcohol impact on taking higher sexual risks than expected by respondent** | We collapsed category “Don´t remember/don´t know” with “Big impact times” as we reasoned that the respondent, who did not remember or did not know what impact alcohol use had on them most likely had big impact. We had 15 persons in the category “Don´t remember/Don´t know “. |
| - No impact | - No and little impact |  |
| - Little impact |  |  |
| - Some impact | - Some impact |  |
| - Big impact | - Big impact and don´t remember/don´t know |  |
| - Don´t remember/don´t know |  |  |
|  | - Not applicable, did not drink |  |
| **Drug use before having sex (past 6 months)** | **Drug use before having sex (past 6 months)** | We collapsed category “Don´t remember/don´t know” with “Any use” as we reasoned that the respondent, who did not remember or did not know whether they used any drugs most likely used them. We had 9 persons in the category “Don´t remember/Don´t know “. |
| - No | - No |  |
| - Sometimes | - Any use and don´t remember/don´t know |  |
| - Several times |  |  |
| - Don´t remember/don´t know |  |  |
| **Drug impact on taking higher sexual risks than expected by respondent (n=268)*** | **Drug impact on taking higher sexual risks than expected by respondent** | We collapsed category “Don´t remember/don´t know” with “Big impact times” as we reasoned that the respondent, who did not remember or did not know what impact drug use had on them most likely had big impact. We had 12 persons in the category “Don´t remember/Don´t know “. |
| - No impact | - No and little impact |  |
| - Little impact |  |  |
| - Some impact | - Some impact |  |
| - Big impact | - Big impact and don´t remember/don´t know |  |
| - Don´t remember/don´t know |  |  |
| For men only (n=1436):  **Have ever made woman unintentionally pregnant** | For men only (n=1436):  **Have ever made woman unintentionally pregnant** |  |
| - No | - No |  |
| - Once | - At least once |  |
| - Several times |  |  |
| - Don´t remember/don´t know | - Don´t remember/don´t know |  |
| For women only (n=1378):  **Current contraception method** | For women only (n=1378):  **Current contraception method** |  |
| - No birth control, other reason | No contraceptives |  |
| - Combined oral contraceptive pills | - Any hormonal contraceptive |  |
| - Progesterone-only pills |  |  |
| - Contraceptive pole |  |  |
| - Contraceptive ring |  |  |
| - Hormonal loop or coil |  |  |
| - Diaphragm |  |  |
| - No birth control, wish pregnancy | - Wish pregnancy |  |
| - Condom | - Barrier method |  |
| - Calendar method/interrupted intercourse | - Calendar method |  |
| - Sterilisation | - Sterilised |  |
| **Use of emergency contraceptive pills (lifetime)** | **Use of emergency contraceptive pills (lifetime)** |  |
| - No | - No |  |
| - Yes, once | - At least once |  |
| - Yes, several times |  |  |
| **Did induced abortion (lifetime)** | **Did induced abortion (lifetime)** |  |
| - No | - No |  |
| - Yes, once | - At least once |  |
| - Yes, several times |  |  |

* - Follow-up question if answered previous question.

## Table 3S. Latent class conditional response probabilities across levels of each variable for men (n= 1,436)*

|  | **Class 1: Mixed steady and non-steady partnerships, low substance use** | **Class 2: Steady partnerships with/without concurrent partners** | **Class 3: Non-steady partnerships with many partners, condom users** | **Class 4: Non-steady partnerships with many partners, condom non-users** |
| --- | --- | --- | --- | --- |
|  | N=110  (8%) | N=441  (30%) | N=601  (42%) | N=284  (20%) |
| **Current Main Testing Reason for Chlamydia** | | | | |
| Safety and new partner requested and Other | 0.512 | 0.551 | 0.406 | 0.367 |
| **Sex with casual partner** | **0.109** | **0.116** | **0.233** | **0.208** |
| Partner has chlamydia | 0.163 | 0.185 | 0.197 | 0.212 |
| Symptoms | 0.217 | 0.148 | 0.164 | 0.213 |
| **Steady and Concurrent relationship** | | | | |
| **No steady partner and no/missing concurrent** | **0.483** | **0.075** | **0.819** | **0.727** |
| Yes steady partner and no concurrent | 0.227 | 0.432 | 0.024 | 0.025 |
| Yes steady partner and yes concurrent | 0.172 | 0.453 | 0.034 | 0.112 |
| Yes steady and missing concurrent | 0.009 | 0.023 | 0.008 | 0.012 |
| Missing information on steady partnership | 0.108 | 0.016 | 0.116 | 0.125 |
| **Number of Sexual Partners (past 12 months)** | | | | |
| 0-2 partners | 0.522 | 0.354 | 0.158 | 0.097 |
| 3-5 partners | 0.315 | 0.432 | 0.390 | 0.440 |
| **≥6 partners** | **0.163** | **0.214** | **0.452** | **0.464** |
| **Type of Last Sexual Partner** | | | | |
| Steady partners | 0.415 | 0.912 | 0.015 | 0.040 |
| Recurrent partners | 0.347 | 0.028 | 0.416 | 0.449 |
| **Casual unknown partners** | **0.097** | **0.000** | **0.267** | **0.183** |
| **Casual known partners** | **0.076** | **0.020** | **0.188** | **0.172** |
| Other possible partners | 0.065 | 0.040 | 0.114 | 0.156 |
| **Condom with New/Casual partner** | | | | |
| **Never and seldom** | **0.264** | **0.286** | **0.050** | **0.876** |
| Often and always | 0.736 | 0.714 | 0.950 | 0.124 |
| **Condom Responsibility** | | | | |
| **Never and often not** | **0.119** | **0.115** | **0.000** | **0.495** |
| Sometimes | 0.408 | 0.455 | 0.409 | 0.456 |
| Always | 0.473 | 0.429 | 0.591 | 0.049 |
| **Alcohol use before having sex (past 6 months)** | | | | |
| No | 1.000 | 0.000 | 0.000 | 0.000 |
| Sometimes | 0.000 | 0.372 | 0.291 | 0.194 |
| **Several times and don´t remember/don´t know** | **0.000** | **0.628** | **0.709** | **0.806** |
| **Alcohol impact** | | | | |
| No and little impact | 0.047 | 0.529 | 0.338 | 0.336 |
| Some impact | 0.002 | 0.312 | 0.466 | 0.438 |
| **Big impact and Do not remember/don´t know** | **0.000** | **0.159** | **0.197** | **0.226** |
| Not applicable, did not drink | 0.951 | 0.000 | 0.000 | 0.000 |
| **Drug use before having se (past 6 months)** | | | | |
| No use | 0.964 | 0.901 | 0.908 | 0.804 |
| **Any use and Do not remember/ don´t know** | **0.036** | **0.099** | **0.092** | **0.196** |
|  |  |  |  |  |

*- The items marked bold are presented in the main text of the article.

## Table 4S. Latent class conditional response probabilities across levels of each variable for women (n= 1,378)*

|  | **Class 1: Mixed steady and non-steady partnerships, low substance use** | **Class 2: Steady partnership with/without concurrent partners** | **Class 3: Non-steady partnerships with many partners** |
| --- | --- | --- | --- |
|  | N=134  (10%) | N=441  (32%) | N=803  (58%) |
| **Current Main Testing Reason for Chlamydia** | | | |
| Safety and new partner requested and Other | 0.507 | 0.527 | 0.409 |
| **Sex with casual partner** | **0.191** | **0.120** | **0.310** |
| Partner has chlamydia | 0.139 | 0.160 | 0.117 |
| Symptoms | 0.164 | 0.193 | 0.163 |
| **Steady and Concurrent relationship** | | | |
| **No steady partner and no/missing concurrent** | **0.541** | **0.078** | **0.897** |
| Yes steady partner and no concurrent | 0.259 | 0.373 | 0.000 |
| Yes steady partner and yes concurrent | 0.146 | 0.486 | 0.025 |
| Yes steady and missing concurrent | 0.016 | 0.044 | 0.000 |
| Missing information on steady partnership | 0.037 | 0.019 | 0.078 |
| **Number of Sexual Partners (past 12 months)** | | | |
| 0-2 partners | 0.580 | 0.376 | 0.160 |
| 3-5 partners | 0.377 | 0.455 | 0.528 |
| **≥6 partners** | **0.042** | **0.169** | **0.312** |
| **Type of Last Sexual Partner** | | | |
| Steady partners | 0.565 | 0.872 | 0.004 |
| Recurrent partners | 0.227 | 0.054 | 0.536 |
| **Casual unknown partners** | **0.042** | **0.004** | **0.155** |
| **Casual known partners** | **0.089** | **0.020** | **0.177** |
| Other possible partners | 0.076 | 0.049 | 0.128 |
| **Condom with New/Casual partner** | | | |
| **Never and seldom** | **0.145** | **0.239** | **0.329** |
| Often and always | 0.855 | 0.761 | 0.671 |
| **Condom Responsibility** | | | |
| **Never and often not** | **0.147** | **0.243** | **0.272** |
| Sometimes | 0.431 | 0.501 | 0.490 |
| Always | 0.422 | 0.256 | 0.239 |
| **Alcohol use before having sex (past 6 months)** | | | |
| No | 0.900 | 0.000 | 0.000 |
| Sometimes | 0.099 | 0.390 | 0.345 |
| **Several times and don´t remember/ don´t know** | **0.001** | **0.610** | **0.655** |
| **Alcohol impact** | | | |
| No and little impact | 0.494 | 0.510 | 0.390 |
| Some impact | 0.000 | 0.356 | 0.422 |
| **Big impact and Do not remember/ don´t know** | **0.000** | **0.134** | **0.188** |
| Not applicable, did not drink | 0.506 | 0.000 | 0.000 |
| **Drug use before having se (past 6 months)** | | | |
| No use | 0.993 | 0.904 | 0.906 |
| **Any use and Do not remember/ don´t know** | **0.007** | **0.096** | **0.094** |

* - The items marked bold are presented in the main text of the article.

# **Table 5S. Fit statistics of the latent class models for men (n= 1,436) and women (n= 1,378).**

|  | **Men** | | | **Women** | | |
| --- | --- | --- | --- | --- | --- | --- |
|  | **Akaike Information Criteria** | **Bayesian Information Criteria** | **Entropy** | **Akaike Information Criteria** | **Bayesian Information Criteria** | **Entropy** |
| **2-class model** | 24177.84 | 24404.44 | 0.78 | 22801.92 | 23026.74 | 0.84 |
| **3-class model** | 23635.19 | 23956.64 | 0.87 | **22180.07** | **22504.23** | **0.91** |
| **4-class model** | **23111.93** | **23538.77** | **0.85** | Not feasible | Not feasible | Not feasible |
| **5-class model** | 23014.18 | 23535.88 | 0.82 | Not feasible | Not feasible | Not feasible |
| **6-class model** | Not feasible | Not feasible | Not feasible | Not feasible | Not feasible | Not feasible |

# **Table 6S. Probabilities of latent class memberships by covariates for men** **(n= 1,436)**

|  | **Class 1: Mixed steady and non-steady partnerships, low substance use**  (N=110) | **Class 2: Steady partnership with/without concurrent partners**  (N=441) | **Class 3. Non-steady partnerships with many partners, condom users**  (N=601) | **Class 4: Non- steady partnerships with many partners, condom non-users**  (N=284) |
| --- | --- | --- | --- | --- |
| **Age group** |  |  |  |  |
| 20-24 | 26.36 | 24.04 | 24.13 | 30.63 |
| 25-29 | 37.27 | 44.90 | 42.93 | 45.42 |
| 30-34 | 22.73 | 20.63 | 23.13 | 17.25 |
| 35-40 | 13.64 | 10.43 | 9.82 | 6.69 |
| **Mean age (years)** | 28.5 (±4.7) | 27.9 (±4.4) | 28.0 (±4.4) | 27.1 (±4.1) |
| **Marital status** |  |  |  |  |
| Married and cohabitation | 16.36 | 29.59 | 2.17 | 4.24 |
| Living apart | 13.64 | 35.78 | 1.34 | 4.24 |
| Single | 64.55 | 20.18 | 93.48 | 88.34 |
| other | 5.45 | 14.45 | 3.01 | 3.18 |
| **Got woman unintentionally pregnant** |  |  |  |  |
| No | 66.67 | 66.59 | 66.38 | 51.61 |
| At least once | 28.70 | 27.65 | 28.01 | 39.78 |
| Don't remember/don´t know | 4.63 | 5.76 | 5.60 | 8.60 |

# **Table 7S. Probabilities of latent class memberships by covariates for women (n= 1,378).**

|  | **Class 1: Mixed steady and non-steady partnerships, low substance use**  (N=134) | **Class 2. Steady partnership with/without concurrent partners**  (N=441) | **Class 3: Non-steady partnerships with many partners** (N=803) |
| --- | --- | --- | --- |
| **Age group** |  |  |  |
| 20-24 | 29.10 | 31.07 | 35.99 |
| 25-29 | 32.84 | 46.49 | 41.59 |
| 30-34 | 23.13 | 14.29 | 15.57 |
| 35-40 | 14.93 | 8.16 | 6.85 |
| **Mean age (years)** | 28.4 (±5.0) | 26.9 (±4.3) | 26.7 (±4.2) |
| **Marital status** |  |  |  |
| Married and cohabitation | 9.85 | 28.90 | 1.00 |
| Living apart | 16.67 | 40.14 | 0.87 |
| Single | 62.88 | 17.89 | 95.63 |
| other | 10.61 | 13.07 | 2.50 |
| **Contraceptives** |  |  |  |
| No contraceptives | 3.31 | 1.17 | 0.90 |
| Any hormonal contraceptive | 34.71 | 38.32 | 27.41 |
| Barrier method | 29.75 | 23.36 | 35.14 |
| Calendar method | 15.70 | 24.77 | 23.55 |
| Wish pregnancy | 16.53 | 11.68 | 12.87 |
| Sterilized | 0.00 | 0.70 | 0.13 |
| **Lifetime use of emergency contraceptive pill** |  |  |  |
| No | 31.06 | 29.95 | 30.59 |
| At least once | 68.94 | 70.05 | 69.41 |
| **Induced abortion (ever)** |  |  |  |
| No | 67.94 | 68.28 | 74.71 |
| At least once | 32.06 | 31.72 | 25.29 |

**Table 8S. Association between latent class membership and repeated testing adjusted for age groups, women**

|  | Short-term outcome: Repeated testing during past 12 months^&^  N= 1,249 | | | Long-term outcome: Repeated lifetime testing^*, #^  N=1,353 | | |
| --- | --- | --- | --- | --- | --- | --- |
| Latent classes | adjOR ^‡^ | 95% CI | P-value | adjOR ^‡^ | 95% CI | P-value |
| Class 1: Mixed steady and non-steady partnerships, low substance use | Reference | | | Reference | | |
| Class 2: Steady partnerships with/without concurrent partners | 1.09 | 0.72-1.65 | 0.679 | 1.26 | 0.83-1.91 | 0.282 |
| Class 3: Non-steady partnerships with many partners | **1.72** | **1.16-2.54** | **0.007** | **1.85** | **1.24-2.76** | **0.002** |

& - Test for linear trend (P =0.0071);

* - Test for linear trend (P =0.0024);

# - Brant test (P>0.05), the proportional odds assumption not violated.

‡ - adjOR, Odds Ratio adjusted for age groups

**Table 9S. Association between latent class membership and repeated testing adjusted for age groups, men**

|  | Short-term outcome: Repeated testing during past 12 months^&^  N=1,136 | | | Long-term outcome: Repeated lifetime testing^*, #^  N=1,410 | | |
| --- | --- | --- | --- | --- | --- | --- |
|  | adjOR ^‡^ | 95% CI | P-value | adjOR ^‡^ | 95% CI | P-value |
| Class 1: Mixed steady and non-steady partnerships, low substance use | Reference | | | Reference | | |
| Class 2: Steady partnerships with/without concurrent partners | 0.99 | 0.59-1.67 | 0.975 | 1.44 | 0.94 -2.18 | 0.091 |
| Class 3: Non-steady partnerships with many partners, condom users | 1.60 | 0.97-2.65 | 0.065 | **2.26** | **1.50 - 3.40** | **0.000** |
| Class 4: Non-steady partnerships with many partners, condom non-users | 1.32 | 0.77-2.26 | 0.306 | **3.03** | **1.93 -4.74** | **0.000** |

& - Test for linear trend (P =0.1135);

* - Test for linear trend (P <0.0001);

# - Brant test (P >0.05), the proportional odds assumption not violated.

‡ - adjOR, Odds Ratio adjusted for age groups

**Table 10S. Association between latent class membership and present chlamydia infection, and repeated lifetime chlamydia infection adjusted for age groups, men**

|  | Short-term outcome: Present Chlamydia infection ^1,&^  N= 1,436 | | | Long-term outcome: Repeated lifetime Chlamydia infection ^2^  N=1,121 | | | | | |
| --- | --- | --- | --- | --- | --- | --- | --- | --- | --- |
|  |  | | | Once versus never ^*^ | | | 2 and more times versus once ^#^ | | |
|  | adjOR ‡ | 95% CI | P-value | adjOR ‡ | 95% CI | P-value | adjOR ‡ | 95% CI | P-value |
| Class 1: Mixed steady and non-steady partnerships, low substance use | Reference | | | Reference | | | Reference | | |
| Class 2: Steady partnerships with/without concurrent partners | 1.87 | 0.82-4.27 | 0.135 | 1.80 | 0.99 - 3.23 | 0.051 | 0.81 | 0.29 - 2.28 | 0.688 |
| Class 3: Non-steady partnerships with many partners, condom users | 2.16 | 0.97-4.83 | 0.061 | **1.84** | **1.03 - 3.26** | **0.038** | 1.12 | 0.42 - 2.95 | 0.825 |
| Class 4: Non-steady partnerships with many partners, condom non-users | **3.03** | **1.32-6.93** | **0.009** | **2.54** | **1.39 - 4.64** | **0.002** | 2.52 | 0.94 - 6.70 | 0.065 |

1 – Ordered logistic regression model;

2 – Multinomial logistic regression model;

& - Test for linear trend (P=0.0069);

* - Test for linear trend (P=0.0026);

# - Test for linear trend (P= 0.0433).

‡ - adjOR, Odds Ratio adjusted for age groups

**Table 11S. Association between latent class membership and present chlamydia infection, and repeated lifetime chlamydia infection adjusted for age groups, women**

|  | Short-term outcome: Present Chlamydia infection *  N= 1,378 | | | Long-term outcome: Repeated lifetime Chlamydia infection ^#, *^  N=1,285 | | |
| --- | --- | --- | --- | --- | --- | --- |
|  | adjOR ^‡^ | 95% CI | P-value | adjOR ^‡^ | 95% CI | P-value |
| Class 1: Mixed steady and non-steady partnerships, low substance use | Reference | | | Reference | | |
| Class 2: Steady partnerships with/without concurrent partners | 1.47 | 0.69-3.11 | 0.315 | 1.28 | 0.84-1.96 | 0.252 |
| Class 3: Non-steady partnership with many partners | 1.28 | 0.62-2.65 | 0.501 | 1.28 | 0.86-1.91 | 0.232 |

* - Test for linear trend (P>0.05);

# - Brant test (P=0.198), the proportional odds assumption not violated.

‡ - adjOR, Odds Ratio adjusted for age groups

**References**

1. Aghaizu A, Reid F, Kerry S, Hay PE, Mallinson H, Jensen JS et al. Frequency and risk factors for incident and redetected Chlamydia trachomatis infection in sexually active, young, multi-ethnic women: a community based cohort study. Sexually transmitted infections. 2014;90(7):524-8. doi:10.1136/sextrans-2014-051607.

2. Klovstad H, Grjibovski A, Aavitsland P. Population based study of genital Chlamydia trachomatis prevalence and associated factors in Norway: a cross sectional study. BMC infectious diseases. 2012;12:150. doi:10.1186/1471-2334-12-150.

3. Learner ER, Torrone EA, Fine JP, Pence BW, Powers KA, Miller WC. Chlamydia Prevalence Trends Among Women and Men Entering the National Job Training Program From 1990 Through 2012. Sexually transmitted diseases. 2018;45(8):554-9. doi:10.1097/OLQ.0000000000000798.

4. Navarro C, Jolly A, Nair R, Chen Y. Risk factors for genital chlamydial infection. The Canadian journal of infectious diseases = Journal canadien des maladies infectieuses. 2002;13(3):195-207. doi:10.1155/2002/954837.

5. Shiely F, Hayes K, Horgan M. Comparison of risk factors for prevalent sexually transmitted infections based on attendees at two genitourinary medicine clinics in Ireland. International journal of STD & AIDS. 2014;25(1):29-39. doi:10.1177/0956462413491732.

6. Sonnenberg P, Clifton S, Beddows S, Field N, Soldan K, Tanton C et al. Prevalence, risk factors, and uptake of interventions for sexually transmitted infections in Britain: findings from the National Surveys of Sexual Attitudes and Lifestyles (Natsal). Lancet. 2013;382(9907):1795-806. doi:10.1016/S0140-6736(13)61947-9.

7. Novak M, Novak D. Risk factors for Chlamydia trachomatis infection among users of an internet-based testing service in Sweden. Sexual & reproductive healthcare : official journal of the Swedish Association of Midwives. 2013;4(1):23-7. doi:10.1016/j.srhc.2012.11.004.

8. Velicko I, Ploner A, Sparen P, Marions L, Herrmann B, Kuhlmann-Berenzon S. Sexual and testing behaviour associated with Chlamydia trachomatis infection: a cohort study in an STI clinic in Sweden. BMJ open. 2016;6(8):e011312. doi:10.1136/bmjopen-2016-011312.

9. Carre H, Lindstrom R, Boman J, Janlert U, Lundqvist L, Nylander E. Asking about condom use: a key to individualized care when screening for chlamydia. International journal of STD & AIDS. 2011;22(8):436-41. doi:10.1258/ijsa.2011.010481.

10. Edgardh K, Kuhlmann-Berenzon S, Grunewald M, Rotzen-Ostlund M, Qvarnstrom I, Everljung J. Repeat infection with Chlamydia trachomatis: a prospective cohort study from an STI-clinic in Stockholm. BMC public health. 2009;9:198. doi:10.1186/1471-2458-9-198.
